# Predictors of Decision-Making Regarding Endocrine Therapy in Breast Cancer Survivors: A Systematic Review

**DOI:** 10.3390/jcm15020858

**Published:** 2026-01-21

**Authors:** Beatriz Mesquita, Ana Bártolo, Sónia Remondes-Costa, Joana Carreiro, Susana Cardoso

**Affiliations:** 1 Department of Education and Psychology, University of Trás-os-Montes e Alto Douro, 5000-801 Vila Real, Portugal; biatrizmesquita@gmail.com; 2 CINTESIS.UPT@RISE-Health, Portucalense University, 4200-072 Porto, Portugal; ana.bartolo@upt.pt; 3 Research Center in Sports Sciences, Health Sciences and Human Development (CIDESD), Department of Education and Psychology, University of Trás-os-Montes e Alto Douro, 5000-801 Vila Real, Portugal; costas@utad.pt; 4 Department of Social Sciences and Behavior, University of Maia, 4475-690 Maia, Portugal; jcarreiro@umaia.pt; 5 Laboratory of Neuropsychophysiology, Faculty of Psychology and Education Sciences, University of Porto, 4200-135 Porto, Portugal

**Keywords:** breast cancer, decision-making, quality of life, endocrine therapy

## Abstract

**Background/Objectives**: Endocrine therapy (ET) is a common treatment for hormone-dependent breast cancer and is associated with a significant reduction in recurrence and mortality rates. However, the decision to initiate endocrine therapy is a critical and often distressing juncture for patients. The need to weigh its survival benefits against the potential burden of side effects, including mood changes, pain, muscle stiffness, and fatigue, can render this decision-making phase a source of significant distress. The present systematic review aimed to identify and synthesize the sociodemographic and psychosocial predictors of the decision-making process related to ET adherence among women with breast cancer. **Methods**: A systematic literature search was conducted in three electronic databases—PubMed Central, ProQuest, and Scopus—to identify studies examining the association between sociodemographic and psychosocial factors and the decision-making process regarding ET among women with breast cancer. Inclusion criteria encompassed cross-sectional studies published between 2000 and 2025. Data were extracted and analyzed to identify recurring predictors across studies. The findings were synthesized through a narrative synthesis. **Results**: Twelve cross-sectional studies met the inclusion criteria, comprising a total of 8510 women diagnosed with breast cancer and undergoing ET. Ten studies (83%) identified sociodemographic variables—such as age, marital status, educational level, and ethnicity—as significant predictors of decision-making. Moreover, nine studies (75%) reported psychosocial factors, including quality of life (QoL), fear of progression, infertility concerns, and social support, as influential in the decision to initiate or continue ET. Specifically, the decision to adhere to ET is generally supported by younger age, higher education, better perceived quality of life, and greater social support. Conversely, it is hindered by lower income, lower education, fertility concerns related to marital status, and diminished quality of life. **Conclusions:** The findings of this review indicate that both sociodemographic and psychosocial factors play key roles in shaping women’s decisions regarding adherence to ET. Understanding these predictors can facilitate decision-making and inform the development of targeted interventions aimed at improving treatment adherence and supporting patient-centered care in breast cancer treatment. The focus on decision-making processes, rather than on adherence rates, is what distinguishes this review from other systematic reviews.

## 1. Introduction

Breast cancer remains the most common malignancy among women worldwide, accounting for nearly two million new cases diagnosed among women aged 20 to 69 years in 2022 and over 400,000 deaths annually [1]. This high disease burden underscores the continued need for effective prevention, treatment, and survivorship strategies, as well as a deeper understanding of the factors influencing therapeutic decision-making throughout the cancer care continuum.

A key component in the management of hormone receptor–positive breast cancer, which accounts for approximately two-thirds of all cases, is Endocrine Therapy (ET). The decision to include ET in the treatment plan is made jointly by the patient and the oncologist, based on clinical factors such as initial risk assessment, lymph node involvement, and tumor histology. ET options, including tamoxifen and aromatase inhibitors, aim to block the interaction between estrogens and the cellular pathways responsible for hormone-dependent tumor growth [2].

Given the central role of ET, there is a growing research interest in understanding the therapeutic decision-making process, particularly the psychosocial and clinical factors influencing women’s choices. Such decisions in oncology often involve navigating complex options with uncertain outcomes and significant emotional consequences [3,4].

This complexity is heightened because, despite its proven efficacy in reducing recurrence and mortality, endocrine therapy (ET) is frequently associated with side effects, such as hot flashes, myalgia, osteoporosis, thromboembolic events, and an increased risk of uterine cancer, which can pose considerable challenges to long-term treatment [5]. Consequently, the choice to adhere to ET is shaped not only by clinical indicators but also by a broader range of sociodemographic, medical, and psychosocial determinants. Key factors include ethnicity, financial status, comorbidities, tumor stage, depressive symptoms, fear of recurrence, and the perceived impact of symptoms on quality of life [5,6,7,8].

Empirical evidence supports the influence of psychological and physical factors on adherence. For instance, Mao et al. [9] found that affective disturbances and chronic pain disorders were negatively associated with continuation of ET, while therapy-related symptoms further exacerbated treatment discontinuation. Such circumstances place patients in a state of uncertainty about both short- and long-term outcomes—a condition referred to as decisional conflict. This conflict is particularly prevalent in breast cancer treatment, where therapeutic options carry major implications and may elicit feelings of doubt or guilt regarding the choices made.

High levels of decisional conflict have been shown to significantly affect both treatment decision-making and emotional adjustment among women with cancer [10]. De Morgan et al. [11] reported that 47% of women with ductal carcinoma in situ (DCIS) experienced decisional conflict during treatment choice. Moreover, concerns associated with ET adherence often persist over time [12].

Given that the continuation of adjuvant therapy is crucial to maximizing treatment efficacy and reducing recurrence risk, understanding the determinants of adherence is a clinical and psychosocial priority. Despite its importance, treatment interruptions remain common [12]. While previous reviews have explored treatment decision aids for early-stage breast cancer [13] or synthesized the determinants of adherence to adjuvant ET [14], the factors specifically influencing women’s decision-making regarding ET initiation and continuation remain insufficiently understood. In particular, the relationship between sociodemographic and psychosocial variables and the decision-making process itself—beyond adherence rates—has not yet been systematically reviewed.

It is worth noting that this study is theoretically grounded in the framework of shared decision-making. This process occurs when healthcare professionals and patients collaborate to decide which tests and treatments are best suited for the patient.

The present study is a systematic review of the literature on sociodemographic and psychosocial predictors of the decision-making process regarding endocrine therapy for breast cancer. By systematically identifying and analyzing these factors, this review aims to provide a comprehensive understanding of how women navigate this critical treatment choice. The findings are intended to inform strategies that enhance shared decision-making and support long-term adherence. The key distinction of this review is its focus on the decision-making process itself, rather than on adherence rates.

## 2. Materials and Methods

This review was conducted following the Preferred Reporting Items for Systematic Reviews and Meta-Analyses (PRISMA) 2020 guidelines [15]. It was registered in the International Prospective Register of Systematic Reviews (PROSPERO) under registration number CRD42024515840.

### 2.1. Eligibility Criteria

The Population, Intervention, Comparison, and Outcomes (PICO) framework guided the definition of inclusion criteria. This review included studies involving women diagnosed with hormone-dependent breast cancer for at least six months, from stage 0 (DCIS) to stage IIA, who had undergone ET. Only cross-sectional studies that examined predictive relationships between sociodemographic variables (e.g., age, educational level, marital status, among others) and psychosocial variables (e.g., social support, quality of life [QoL], concerns about infertility) and therapeutic decision-making were included, provided they were published in English and/or Portuguese. Review articles, abstracts, conference proceedings, and academic dissertations/theses were excluded from this study.

### 2.2. Search Strategy

A systematic review was conducted using three electronic databases: PubMed Central, ProQuest, and Scopus. The literature search was originally performed between 29 September and 6 October 2023, and subsequently updated on 4 September 2025, to ensure the inclusion of the most recent studies. The search strategy used the keywords “decision-making” AND “breast cancer” (OR “breast neoplasms” OR “breast carcinoma”) AND “endocrine therapy” (OR “hormonal therapy”), limited to publications from January 2000 to September 2025. Terms were searched in English, Spanish, and Portuguese, using the Boolean operators “AND” and “OR” to combine concepts. Filters were applied to restrict the results to empirical research articles and exclude clinical trials, systematic reviews, meta-analyses, and longitudinal designs. In addition, a complementary manual search was performed by reviewing the reference lists of all included studies to identify any additional eligible publications not captured by the database search. The complete and reproducible search strategies for each database are provided in Supplementary Table S2.

### 2.3. Selection and Data Extraction Process

All authors jointly defined the objective of the systematic review, including the search strategy, and inclusion and exclusion criteria. B.M. conducted the database searches. B.M. and S.C. independently screened titles and abstracts and assessed the full texts of potentially eligible studies. Study quality was independently evaluated using the JBI critical appraisal tools by B.M. and S.C. Any discrepancies were resolved through discussion; however, no disagreements arose, and the involvement of a third reviewer was not required. The Rayyan web-based tool was used to facilitate and manage the screening process (Rayyan, 1 Broadway, 14º andar, Cambridge, MA, USA, EUA, web-based platform). For each included study, the following data were extracted: author(s), country, study design, sample size, participants’ mean age (M) and standard deviation (SD), type of Endocrine Therapy administered, assessment time points, predictors of decision-making, and the direction of the reported effects. The heterogeneity across studies in terms of design, population, and outcome measures precluded a quantitative meta-analysis. Consequently, a narrative and descriptive synthesis was performed, focusing on the direction and consistency of the reported associations.

### 2.4. Study Quality

Two reviewers (B.M. and S.C.) independently assessed the quality of the eligible studies using the Joanna Briggs Institute (JBI) critical appraisal checklists for cross-sectional studies, from the JBI Statistics Assessment and Review Instruments. Each checklist item was rated as “yes”, “no”, “unclear”, or “not applicable”. Consensus was reached through discussion with a third author when necessary. The assessment of the methodological quality of the studies was based on the information available in the articles. The JBI quality assessment was used to guide the interpretation of the results by facilitating the identification of potential methodological limitations across studies, without excluding any studies based on quality criteria.

## 3. Results

### 3.1. Study Selection

**Figure 1:**
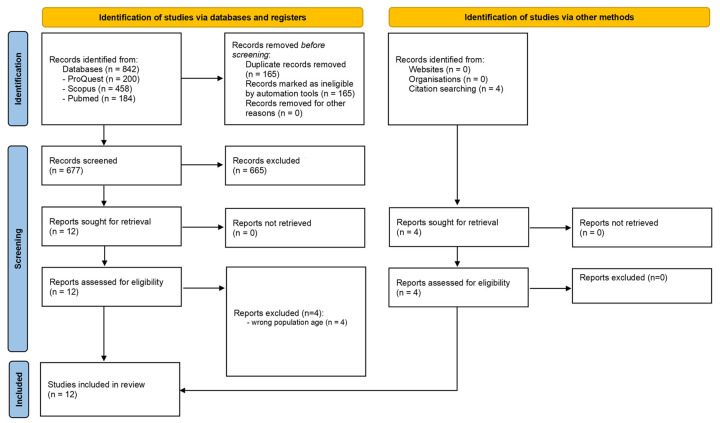
PRISMA Selection Process Flow Chart.

A total of 842 potentially relevant articles were identified in the electronic databases. After removing duplicates, 677 articles were screened by title and abstract, but 665 did not meet the eligibility criteria. The full texts of 12 potentially eligible articles were retrieved, but only eight met the criteria. Subsequently, four additional studies were identified through previous citations and included in the review. In total, 12 articles were deemed eligible for this review. The PRISMA flow diagram of the search and study selection process, including the reasons for article exclusion, is presented in Figure 1.

### 3.2. Study Characteristics

This review included 12 cross-sectional studies conducted in USA (*n* = 3), China (*n* = 2), The Netherlands (*n* = 2), France (*n* = 2), Australia (*n* = 1), USA and Canada (*n* = 1), and Canada (*n* = 1) see Table S1. These studies were published between 2011 and 2023 and recruited a total of 8510 women diagnosed with breast carcinoma who were offered or had undergone ET. The sample sizes of the included studies ranged from 241 [16] to 4211 [17] (see Table 1). A considerable number of articles (*n* = 9) focused on examining the influence of sociodemographic variables (e.g., educational level, age, occupational group, marital status, among others) on the decision-making process [16,18,19,20,21,22,23,24]. In parallel, some studies (*n* = 8) aimed to explore the influence of psychosocial variables (e.g., QoL [depression and anxiety], concerns about infertility, social support, perceived physical symptoms, fear of recurrence, among others) on treatment-related decision-making [6,18,19,20,21,25,26]. It was also possible to identify other outcomes explored in the included studies. In this respect, a total of seven studies sought to investigate data relating to knowledge/informational needs (e.g., beliefs about medication intake, perceived self-efficacy, perception of the different attributes of ET, patient involvement in decision-making, among others) [6,16,19,24,27].

### 3.3. Participant Characteristics

The age of the patients ranged from 36 years [21,23] to 60.13 years [6] (M = 48.1), with the SD impossible to calculate due to missing data in some of the studies. All participants were female and had either undergone or been offered ET (100%). Despite the variety of outcomes explored, all studies (n = 12) aimed to identify which sociodemographic and psychosocial variables predicted the decision-making process regarding endocrine treatment.

A total of four of the studies analysed did not provide any information regarding the marital status of the participants [6,19,22,25]. According to the remaining studies, most participants were married or cohabiting (72–97.1%). In the studies in which the race/ethnicity variable was considered [6,19,20,21,23], many women were non-Hispanic white (90–48.3%). About educational level, most participants had completed secondary education or higher [17,26], and a large proportion of the investigations included women with a high level of education [6,16,18,19,22]. Most women were employed. Among these, a portion reported working in manual occupations (45–54.3%) [17,22], although the remaining studies did not report the nature of professional occupation (See Table 2). An important factor to analyse concerns the stage of disease at diagnosis. In the studies where this variable was examined, most patients were at stage II breast cancer (44–45.7%) [6,17,19,25,26]. Only one study [6] reported that many women were at stage I (69.5%). One study did not provide any information regarding this variable [25]. Of the three studies that aimed to explore concerns about infertility, 58% of women reported being less concerned [23], and 55% stated that they were not at all concerned about these issues [20]. In one study, a considerable proportion of women (79%) expressed concern about discussing infertility issues with their physician before starting ET [21].

### 3.4. Predictive Factors in the Decision-Making Process

The synthesis and analysis of the results of the various studies indicated that 67% of the studies identified age as a significant predictor in the decision-making process [6,16,18,19,26,27]. Likewise, educational level was identified as a predictor in 44% of the studies [17,18,22,23], marital status in 33% [6,18,27], employment status in 20% [17,22] [although without statistical significance], and ethnicity as a significant predictor in 22% of the studies analysed [6,27]. Regarding the influence of psychosocial variables on decision-making, we found that received/perceived social support was considered by 50% [18,24,25,27] as a significant predictor. QoL (e.g., anxiety, depression, psychological domain [mental health]) was referred to as a predictor in 38% of the studies [8,25]. Similarly, concerns about infertility and the need for prior discussion about it were identified as predictors in two studies (25%) [25,27]. In 38% of the studies, variables relating to clinical and physical symptoms and concerns about their onset [6,18,25] were considered significant predictors. Finally, two articles (25%) identified fear of disease progression, perceived disease risk, and fear of death (20%) as significant predictors in the decision-making process [19,25]. Although the overall aim of this systematic review was to explore the influence of sociodemographic and psychosocial factors, the set of selected and reviewed articles also identified other factors that contributed to the decision-making process regarding endocrine treatment in women with breast carcinoma. A total of seven studies identified knowledge/informational needs as predictive outcomes in the decision-making process regarding ET. Of these, 71% identified that obtaining prior information about ET [18,19,25,26] significantly influenced adherence. Finally, perceived treatment self-efficacy was identified in one study (14%) as a significant predictor [16], as well as perceived need and concerns/knowledge about medication use/ET administration in two of the studies analysed (29%) [8,16]. Table 3 presents the results of studies examining predictors of therapeutic decision-making in the context of breast cancer. In the Direction of effect column, directionality is indicated according to whether the predictor facilitates or hinders the decision-making process. A positive effect denotes factors that facilitate decision-making, whereas a negative effect denotes factors that impede it.

### 3.5. Quality Assessment

Table 4 presents the results of the critical appraisal of the 12 cross-sectional studies included in this systematic review. All the studies analysed had clearly defined inclusion criteria. Most of the studies clearly and objectively described the participants as well as the setting and conditions in which the research was to be conducted. Only one study did not clearly and comprehensively define these aspects [22]. Except for one study, which did not present clear information [18], all others demonstrated a valid and reliable method for measuring the targeted outcomes. Similarly, it was found that all the studies used objective and appropriate criteria to assess and manage the identified outcomes. A total of five studies identified confounding factors that may have influenced both the independent and dependent variables [6,8,17,24,26]. At the same time, three studies did not clarify the existence of such factors [18,25,26], and four studies did not meet the criteria for this classification due to their absence [16,19,20,22]. Regarding the studies that identified confounding factors, only three reported having found strategies to deal with them [17,24,26], five did not make this aspect clear [8,18,24,25,26], and the remainder did not address it, as no confounding factors had been previously identified. Finally, it was observed that eight of the studies presented results that were measured validly and reliably [6,16,17,22,24,25,26], while the remaining four did not make the information clear regarding this aspect [8,18,19,24].

Based on the JBI critical appraisal, four studies [16,17,21,23] were classified as having high methodological quality, as they met all appraisal criteria. The remaining eight studies [6,18,19,20,22,24,25,26] were classified as lower methodological quality due to the presence of several unmet or unclear criteria, mainly related to insufficient information on confounding factors, strategies to control them, or the reliability of outcome measurement. This classification was applied for descriptive purposes, as the JBI tool does not provide a standardized quality scoring system.

## 4. Discussion

The present study aimed to identify the predictors of the decision-making process in women with breast cancer undergoing ET. Overall, the findings from the studies analyzed suggest that the decision to initiate or continue ET is shaped by a multifactorial set of clinical, sociodemographic, and psychosocial factors. This indicates that therapeutic decision-making is inherently complex, reflecting an interplay between personal, interpersonal, and contextual variables that may or may not be within the woman’s control.

The interaction between the perceived advantages and disadvantages of ET and individual patient characteristics holds significant implications for clinical practice. Among sociodemographic predictors, educational level consistently emerged as an influential factor. Several studies [17,22,23,24] demonstrated a positive association between higher educational attainment and treatment adherence, suggesting that greater health literacy facilitates understanding of ET’s purpose and benefits. Conversely, women with lower educational levels appeared more prone to intentional non-adherence, possibly due to limited comprehension of treatment efficacy or difficulty managing side effects. These results highlight the need for oncology teams to communicate treatment information in accessible, patient-tailored ways to mitigate disparities in understanding and adherence.

Age also emerged as a relevant predictor, though findings were mixed. While some studies [16,22] identified older age as a risk factor for reduced adherence, others [20,26] suggested that younger women were more actively engaged in decision-making and more likely to maintain ET. This discrepancy may be explained by differing motivational dynamics: older women may prioritize quality of life and minimizing side effects over the potential long-term survival benefits of ET [24], whereas younger women may value disease control and life expectancy more strongly. Importantly, infertility concerns were found to influence the decisions of younger patients in particular [21], reflecting the emotional and existential dimensions that accompany cancer treatment during reproductive years.

Marital status and ethnicity were additional sociodemographic factors associated with decision-making behavior. Married women often reported greater anxiety about infertility, which in turn contributed to decisions not to initiate or to discontinue treatment [20,21,24]. Ethnic disparities were also evident: women of Black or non-White ethnic backgrounds demonstrated lower rates of ET initiation and persistence [20,21], findings consistent with broader evidence linking minority status to unequal access to healthcare resources and psychosocial support. Likewise, lower income and unstable employment conditions were associated with greater barriers to adherence [22]. These findings underscore the importance of addressing social determinants of health in cancer care and promoting equity-oriented interventions.

Psychosocial factors, particularly quality of life (QoL), played a crucial role in the decision-making process. Several studies [6,25] identified symptoms such as fatigue, hot flashes, sleep difficulties, mood changes, and perceived loss of femininity or attractiveness as significant deterrents to treatment adherence. Reduced QoL was consistently linked to lower motivation to continue ET. Similarly, ref. [26] reported that women with poorer psychological well-being tended to adopt a more passive role in therapeutic decisions. Fear of disease progression also emerged as a key emotional determinant, shaping both adherence and decisional conflict [25].

Social support, particularly from family, friends, and healthcare providers, was another recurrent predictor of adherence. Refs. [18,23] showed that both perceived and received support were strongly associated with treatment persistence, while [20] further demonstrated that shared decision-making involving significant others enhanced initiation of ET. The support provided by medical teams in addressing fertility concerns and clarifying treatment benefits was particularly valued by patients [21], suggesting that patient-centered communication and collaborative relationships can effectively promote adherence.

It is important to acknowledge that contextual differences, such as varying access to healthcare services or specialized fertility counseling, can significantly influence the decision-making process. These factors may alter the relevance or weight of the specific predictors identified in this review, and their integration into clinical practice could help mitigate decisional conflict.

In general, younger age is associated with greater decisional conflict, particularly due to fertility-related concerns. Lower income and being married are linked to increased difficulties in treatment continuation. Likewise, lower perceived quality of life and social support correspond to higher decisional conflict and reduced adherence. Conversely, higher educational attainment is associated with lower decisional conflict and, consequently, greater adherence to therapy.

The reviewed studies also emphasized the importance of informational and educational needs in shaping decisions. Limited knowledge about recurrence risk, side effects, and the benefits of ET was found to perpetuate non-adherence [18,19]. When patients received clear and consistent information from healthcare professionals, adherence improved [6,19]. Practical barriers, such as medication management difficulties reported by approximately half of the participants in [24], further highlight the need for continued medical guidance and psychosocial support during treatment.

While patterns of adherence to endocrine therapy, e.g., [27,28] are increasingly documented, the prior decision-making process for accepting or refusing this long-term treatment remains less understood, and this systematic review highlights this reality.

The patterns identified in our synthesis provide a clear illustration of how this decision process operates in practice, which aligns strongly with the Shared Decision-Making (SDM) framework. This model posits that adherence choices are not merely passive compliance but are influenced by the patient’s active engagement, their perceived balance of benefits and risks, and the quality of their support systems. Our review demonstrates that sociodemographic and psychosocial factors directly modulate this balance; they shape how patients perceive the risks and benefits of ET. This, in turn, fundamentally influences their level of decisional conflict and, ultimately, their commitment to long-term treatment continuation.

### Practical Implications, Limitations, and Future Directions

The identification of predictors of therapeutic decision-making in breast cancer provides critical insights for clinical practice and health policy. Understanding how sociodemographic and psychosocial factors influence treatment choices enables more personalized, empathetic, and effective care. Interventions should therefore focus on improving health literacy, addressing fertility concerns, and reinforcing supportive communication between patients and healthcare professionals. Enhancing psychoeducation and social support systems may substantially improve adherence and long-term outcomes for women undergoing ET.

Despite these promising findings, several limitations should be noted. First, the predominance of cross-sectional designs limits causal inference and precludes the examination of changes in decision-making over time. Second, the review was restricted to studies published in English, Spanish, and Portuguese, which may have led to the exclusion of relevant evidence in other languages and introduced potential language bias. Third, most included studies relied on self-reported data, which may be affected by recall and social desirability bias. Fourth, the limited number of available studies, considering that the generalizability of the findings may be affected by methodological heterogeneity and contextual or cultural variables. Fifth, another factor that could impact the findings of this review and their generalizability is the diversity of treatments the participants may have received. Inconsistencies in treatment modalities could significantly affect study outcomes. Therefore, we suggest this be carefully considered in future research. Finally, the heterogeneity of small samples, assessment tools, and study contexts may have influenced the comparability of results across studies.

Future systematic reviews and empirical studies should expand inclusion criteria to encompass broader age ranges, diverse cultural contexts, additional treatment modalities, and explore regional trends or differences. Longitudinal and mixed-method studies are particularly needed to explore how psychosocial and sociodemographic factors influence decisions over the course of treatment. Furthermore, research should continue to investigate the mechanisms linking these predictors to adherence, informed decision-making, and patient-centered outcomes.

Ultimately, understanding how sociodemographic and psychosocial factors shape decisions about ET is crucial for developing person-centered care models, enhancing personalized clinical communication, and informing healthcare policies that are more responsive to patient needs.

## 5. Conclusions

Adherence to ET among women with breast cancer is shaped by a complex interplay of sociodemographic and psychosocial factors. Younger age, higher educational attainment, better perceived QoL, and greater social support appear to facilitate adherence and reduce decisional conflict. This may be because these factors enhance a patient’s understanding of treatment benefits, self-efficacy, and confidence in managing side effects. Conversely, factors such as lower income, lower education, fertility concerns related to marital status, and diminished QoL can hinder treatment continuation. These barriers likely operate by increasing uncertainty, amplifying perceived treatment burdens, and exacerbating emotional distress. These findings collectively highlight disparities in ET adherence and underscore the potential value of targeted interventions. Patient education programs, psycho-oncology support, and structured decision aids could empower women to make more informed and confident treatment choices. When viewed through the lens of the Shared Decision-Making model, the results suggest that addressing both informational and emotional needs is critical for promoting equitable, patient-centered care. To our knowledge, this is the first review to specifically identify predictors of decision-making among women with breast cancer undergoing ET. Ultimately, by systematically synthesizing the existing evidence, this review provides a foundation for future research and the development of interventions aimed not only at improving adherence but also at supporting confident, well-informed therapeutic decisions from the outset.

## Figures and Tables

**Table 1:** General Characteristics of the Studies Reviewed.

Reference	Country	Study Design	Participants *n* (%) (Women)	Age—*M (SD)*
Cluze et al., 2011 [18]	France	Cross-sectional study	196 women, 100%	37.0 (3.30) */37.3 (3.60) **
Friese et al., 2013 [19]	USA	Cross-sectional study	743 women, 100%	(11.70)
Kadakia et al., 2019 [6]	USA	Cross-sectional study	131 women, 100%	60.13
Liu et al., 2017 [17]	China	Cross-sectional study	4211 women, 100%	48.68 (10.47)
Mercieca-Bebber et al., 2016 [25]	Australia	Cross-sectional study	101 women 100%	56.70
Rosenberg et al., 2023 [20]	Canada	Cross-sectional study	693 women, 100%	36
Sella et al., 2021 [21]	USA and Canada	Cross-sectional study	643 women, 100%	36 (3.90)
Seror et al., 2013 [26]	France	Cross-sectional study	442 women, 100%	36.8 (3.80)
Wang et al., 2013 [22]	China	Cross-sectional study	484 women, 100%	50.1 (11.0)
Wassermann et al., 2019 [23]	USA	Cross-sectional study	384 women, 100%	36 (3.90)
Wouters et al., 2013 [24]	The Netherlands	Cross-sectional study	241 women, 100%	57.2 (10)
Wouters et al., 2014 [16]	The Netherlands	Cross-sectional study	241 women, 100%	57 (10)

Note: * Group that reported premature discontinuation vs. ** Group that reported late discontinuation.

**Table 2:** Sociodemographic characteristics of participants across included studies.

Variable	Category	Percentage Range (%)
Marital status	Not reported	4 studies
	Married or cohabiting	72–97.10
Race/Ethnicity	Non-Hispanic White women	48.30–90
Education level	Secondary education or higher	Majority (>50%)
	High educational attainment	Majority (>50%)
Employment status	Employed	Majority (>50%)
	Manual occupations	45–54.30

**Table 3:** Results of Studies Analyzing Predictors of Therapeutic Decision-Making in the Context of Breast Cancer.

Reference	Type of Therapy	Assessment Moment	Predictors Analysed	Direction Effect	Principles Findings
Cluze et al., 2011 [18]	Endocrine Therapy (ET)	Pós tests	Sociodemographic and clinical variables related to cancer, adherence and continuation of ET, QoL (WHOQOL-BREF), depression (CES-D), informational needs.	↓ social support, ↓ information received = ↑ early/late discontinuation of HRT ↑ menopausal symptoms (fluid retention) = ↑ late discontinuation of ET. (Negative Effect)	Social support (OR = 3.7; 95% CI, 1.14–12.00; *p* = 0.03) and prior information (OR = 0.26; 95% CI, 0.09–0.76; *p* = 0.01) received and menopausal symptoms (fluid retention [*p* = 0.02]) were predictors of decision-making.
Friese et al., 2013 [19]	Endocrine Therapy (ET)	Pós tests	Sociodemographic and clinical variables, beliefs about medication taking (BMQ), concern about recurrence (adapted question).	↑ concern about recurrence = ↑ decision to initiate/continue treatment (Positive Effect) ↓ information received = ↓ decision to initiate treatment (Negative Effect) ↑ age = ↓ decision to continue treatment. (Negative Effect) “Latino” ethnicity = ↑ initiation of therapy ↑ medication adherence = ↑ persistence. (Positive Effect)	Concern about recurrence (OR = 3.54; 95% CI; 1.31–9.56; *p* = 0.03) and information received about ET (OR = 0.24; 95% CI 0.10–0.55; *p* < 0.001) were predictors of decision-making. Latinas (OR = 2.80; 95% CI; 1.08–7.23) were more likely to start ET Age (OR = 0.98; 95% CI; 0.95–1.00; *p* = 0.04), medication use (OR = 4.19; 95% CI; 2.28–7.68; *p* < 0.001) and concerns about side effects were predictors of decision-making.
Kadakia et al., 2019 [6]	Endocrine Therapy (ET)	Pós tests	QV (FACT-ES), willingness to continue receiving TE (adapted question), beliefs about medication use (BMQ-S), anxiety, depression (HADS), risk perception (RRP) and cancer-related concerns (ACS).	↓ QV, ↓ perceived need = non-adherence to prolonged ET (Negative Effect) ↑ belief in taking medication = ↑ willingness to prolong ET. (Positive Effect)	QoL (*p* = 0.045), perceived need (*p* = 0.004), and concerns about taking medication (*p* = 0.0007) were predictors of decision-making. A stronger belief in the need to take medication was the only independent factor associated with the decision to prolong ET (OR = 1.33; 95% CI; 1.09–1.59; *p* = 0.005).
Liu et al., 2017 [17]	Endocrine Therapy (ET), Radiotherapy and Chemotherapy	Pós tests	Educational level and professional group.	Housewives, professionals, private sector employees, and manual workers = ↑ adherence to ET All educational level categories influenced the decision to adhere. (Positive Effect)	Educational level (*p* < 0.001) and professional group (*p* = <0.001) were predictors of decision-making.
Mercieca-Bebber et al., 2016 [25]	Sensitivity sample; Chemotherapy, Endocrine Therapy (ET), or Herceptin	Pós tests	QV (QLQ-C30) and related factors (QLQ-BR23; QLQ-BRECON23; FACT-B) and health literacy (HLQ).	Fear of progression and concern about death = predictive factors in decision-making (Negative Effect) Physical symptoms influenced the decision-making process (Negative Effect)	Physical symptoms such as hair loss (*p* = 0.05), hot flushes (*p* = 0.03) and sadness (*p* = 0.04) were predictors of the decision-making process. Fear of progression (60%) and concerns about death (37%) were also predictors of decision-making.
Rosenberg et al., 2023 [20]	Endocrine Therapy (ET)	Pós tests	Sociodemographic and clinical variables, concerns regarding infertility, previous treatments, and physical and clinical symptoms.	Black women = ↑ probability of non-initiation (Negative Effect) ↓ age, married = ↑ concern about health conditions and infertility = predictors of non-persistence (Negative Effect) Having undergone other treatments and experiencing physical symptoms such as hot flushes and vaginal symptoms = ↓ non-persistence in ET. (Negative Effect)	Age (≤30 vs. 36–40 years, OR = 0.50; 95% CI; 3.16–13.38; *p* = <0.001/31–35 vs. 36–40 years, OR = 3.52; 95% CI; 1.99–6.20; *p* = <0.001), ethnicity (OR = 6.27; 95% CI; 2.41–16.32; *p* = <0.001), marital status (OR = 2.55; 95% CI; 1.31–4.94; *p* = 0.006), education (OR = 0.4; 95% CI; 0.25–0.88; *p* = 0.018), concerns about infertility (OR = 1.93; 95% CI; 1.28–2.92; *p* = 0.002) were predictors of decision-making. Having undergone chemotherapy before (OR = 0.37; 95% CI; 0.21–0.66; *p* = <0.001) and the presence of hot flushes (OR = 0.54; 95% CI; 0.41–0.70; *p* = <0.001) and vaginal symptoms (OR = 0.62; 95% CI; 0.47–0.80; *p* = <0.001) were predictors of decision-making.
Sella et al., 2021 [21]	Endocrine Therapy (ET)	Pós tests	Sociodemographic and clinical variables and concerns related to infertility.	↓ age, non-standard ethnicity, unmarried, nulliparous, and who had a pre-treatment discussion about fertility = ↑ concerns about fertility affecting the decision. Concern about infertility was a significant predictor of non-initiation/non-persistence. (Positive Effect)	Age (*p* = <0.0001), ethnicity (*p* = 0.02), marital status (*p* = <0.0001), concerns about fertility (*p* = <0.0001) and medical support received (*p* = 0.0002) were predictors of decision-making, due to the influence these variables had on concerns about infertility.
Seror et al., 2013 [26]	Endocrine Therapy (ET), Surgery, and Chemotherapy	Pós tests	Sociodemographic variables, QoL (WHOQOL-BREF), depression (CES-D), involvement in decision-making (CPS), persistence in continuing to receive TE, and consumption of psychotropic drugs.	↓ mental health, ↓ age, and unmarried status = predictors of a passive role in decision-making. Involvement in decision-making was strongly influenced by previous experiences in selecting other treatments. (Negative Effect)	QoL (psychological domain) (OR = 2.6; 95% CI; 1.1–6.2; *p* = 0.084), age (OR = 0.4; 95% CI; 0.1–0.8; *p* = 0.022), marital status (*p* = 0.02) and previous experience in selecting treatments (OR = 4.8; 95% CI; 2.7–8.7; *p* = <0.001) were predictors of decision-making.
Wang et al., 2013 [22]	Radiotherapy, chemotherapy, surgery, and endocrine therapy (ET)	Pós tests	Socioeconomic variables.	↑ educational level = ↑ likelihood of adherence to TE Patients’ economic capacity correlates with their treatment choices. (Positive Effect)	Educational level (*p* = 0.031) proved to be a significant predictor of the decision to adhere to ET.
Wassermann et al., 2019 [23]	Endocrine Therapy (ET)	Pós tests	Sociodemographic variables, concerns about fertility, anxiety, depression (HADS), social support, and non-adherence to endocrine therapy.	↓ level of education and ↓ level of social support = ↑ non-adherence. (Negative Effect)	Educational level (OR = 0.50; 95% CI; *p* = 0.04) and social support (OR = 0.98; 95% CI; *p* = 0.01) were found to be predictors of decision-making.
Wouters et al., 2013 [24]	Endocrine Therapy (ET)	Pós tests	Sociodemographic and clinical variables, preferences for different attributes of TE (ACA), beliefs about medication taking (BMQ).	↑ Age, ↑ educational level, and previous treatment had a univariate association with the decision of unintentional non-adherence. Marital status and educational level showed a univariate association with intentional non-adherence. (Negative Effect)	Therapy effectiveness, educational level (β = 0.15, t = 2.3, *p* = 0.02), age (β= −0.26, t = −4.1, *p* = 0.0001), previous treatments (β = 0.19, t = 2.9; *p* = 0.004), marital status (*p* < 0.10) were predictors of decision-making.
Wouters et al., 2014 [16]	Endocrine Therapy (ET)	Pós tests	Sociodemographic variables, TE efficacy, perceived side effects (TMI), perceived self-efficacy (MUSE) and non-adherence (MARS-5).	↑ age, ↑ perception of self-efficacy and ↑ knowledge about treatment, ↓ intentional non-adherence. (Negative Effect)	Age (OR = 0.94; 95% CI; 0.90–0.99; *p* < 0.05), knowledge about medication intake (OR = 0.5; 0.4–0.7; 95% CI; *p* < 0.01), perception of self-efficacy, and knowledge about previous treatment (OR = 0.2; 0.1–0.9; 95% CI; *p* < 0.05) were predictors of decision-making.

**Table 4:** Critical Appraisal of Included Studies Based on JBI Critical Appraisal Tools (checklist for cross-sectional studies).

Author and Year	Were the Criteria for Inclusion in the Sample Clearly Defined?	Were the Subjects and Context of the Study Described in Detail?	Was the Condition Measured in a Valid and Reliable Manner?	Were Objective and Standardised Criteria Used to Measure the Condition?	Were Confounding Factors Identified?	Were Strategies Established to Deal with Confounding Factors?	Were the Results Measured in a Valid and Reliable Manner?
Cluze et al., 2011 [18]	yes	yes	unclear	yes	unclear	unclear	unclear
Friese et al., 2013 [19]	yes	yes	yes	yes	not applicable	not applicable	unclear
Kadakia et al., 2019 [6]	yes	yes	yes	yes	yes	unclear	unclear
Liu et al., 2017 [17]	yes	yes	yes	yes	yes	yes	yes
Mercieca-Bebber et al., 2016 [25]	yes	yes	yes	yes	yes	yes	unclear
Rosenberg et al., 2023 [20]	yes	yes	yes	yes	yes	unclear	yes
Sella et al., 2021 [21]	yes	yes	yes	yes	not applicable	not applicable	yes
Seror et al., 2013 [26]	yes	yes	yes	yes	unclear	unclear	yes
Wang et al., 2013 [22]	yes	unclear	yes	yes	not applicable	not applicable	yes
Wassermann et al., 2019 [23]	yes	yes	yes	yes	yes	yes	yes
Wouters et al., 2013 [24]	yes	yes	yes	yes	unclear	unclear	yes
Wouters et al., 2014 [16]	yes	yes	yes	yes	not applicable	not applicable	yes

## Data Availability

No new data were created or analyzed in this study. Data sharing is not applicable to this article.

## Supplementary Materials

The following supporting information can be downloaded at: https://www.mdpi.com/article/10.3390/jcm15020858/s1, Table S1: PRISMA checklist; Table S2: Search Syntax.
