# Supplementary material for: Predictors of Decision-Making Regarding Endocrine Therapy in Breast Cancer Survivors: A Systematic Review
Source: J Clin Med. 2026 Jan 21;15(2):858. doi: 10.3390/jcm15020858 (PMC12842196; doi:10.3390/jcm15020858)
Supplement: Supplementary file 1 [file jcm-15-00858-s001.zip › jcm-3964102-supplementary table S2.pdf]

Table S2: Search Syntax

Data bases	Search String	Filters Applied
SCOPUS	AND TITLE-ABS-KEY ("breast cancer" OR "breast neoplasms" OR "breast carcinoma") AND TITLE-ABS-KEY ("endocrine therapy" OR "hormonal therapy")	AND PUBYEAR > 1999 AND PUBYEAR < 2025<br><br>AND (LIMIT-TO (DOCTYPE, "ar"))<br><br>AND (LIMIT-TO (SRCTYPE, "j"))<br><br>AND (LIMIT-TO (LANGUAGE, "English") OR LIMIT-TO (LANGUAGE, "Spanish") OR LIMIT-TO (LANGUAGE, "Portuguese"))
PUBMED CENTRAL	("decision making"[Title/Abstract] OR "decision-making"[Title/Abstract]) AND ("breast cancer"[MeSH Terms] OR "breast neoplasms"[Title/Abstract]) AND ("endocrine therapy"[Title/Abstract] OR "hormonal therapy"[Title/Abstract])	Publication date from 2000/01/01 to 2025/09/04;<br><br>Languages: English, Spanish, Portuguese.
PROQUEST	("decision making"[Title/Abstract] OR "decision-making"[Title/Abstract]) AND ("breast cancer"[MeSH Terms] OR "breast neoplasms"[Title/Abstract]) AND ("endocrine therapy"[Title/Abstract] OR "hormonal therapy"[Title/Abstract])	Publication date from 2000/01/01 to 2025/09/04;<br><br>Languages: English, Spanish, Portuguese.
